# Mismatch Repair Universal Screening of Endometrial Cancers (MUSE) in a Canadian Cohort

**DOI:** 10.3390/curroncol28010052

**Published:** 2021-01-15

**Authors:** Jessica Lawrence, Lara Richer, Jocelyne Arseneau, Xing Zeng, George Chong, Evan Weber, William Foulkes, Laura Palma

**Affiliations:** 1 Department of Human Genetics, McGill University, Montreal, QC H3A 0C7, Canada; william.foulkes@mcgill.ca (W.F.); laura.palma@muhc.mcgill.ca (L.P.); 2 Department of Pathology, McGill University Health Centre, Montreal, QC H4A 3J1, Canada; lara.richer@mail.mcgill.ca (L.R.); jocelyne.arseneau@muhc.mcgill.ca (J.A.); 3 Division of Gynecologic Oncology, Departments of Obstetrics and Gynecology, Oncology, and Pathology, McGill University and McGill University Health Centre, Montreal, QC H4A 3J1, Canada; ziggy.zeng@gmail.com; 4 Department of Pathology, Jewish General Hospital, Montreal, QC H3T 1E2, Canada; george.chong@mcgill.ca; 5 Division of Medical Genetics, Department of Specialized Medicine, McGill University Health Centre, Montreal, QC H4A 3J1, Canada; Evan.Weber@MUHC.MCGILL.CA; 6 Cancer Research Program, Research Institute of the McGill University Health Centre, McGill University, Montreal, QC H4A 3J1, Canada

**Keywords:** lynch syndrome, endometrial, universal screening, mismatch repair, genetic, cancer predisposition, Canadian, immunohistochemistry

## Abstract

Background: Approximately 2–6% of endometrial cancers (ECs) are due to Lynch Syndrome (LS), a cancer predisposition syndrome caused by germline pathogenic variants (PVs) affecting the DNA mismatch repair (MMR) pathway. Increasingly, universal tissue-based screening of ECs has been proposed as an efficient and cost-effective way to identify families with LS, though few studies have been published on Canadian cohorts. The purpose of this study was to evaluate the feasibility and overall performance of a universal immunohistochemistry (IHC) screening program for women with EC within a single Canadian university hospital centre. Methods and Results: From 1 October 2015 to 31 December 2017, all newly diagnosed ECs (n = 261) at our centre were screened for MMR protein deficiency by IHC. MMR deficiency was noted in 69 tumours (26.4%), among which 53 had somatic MLH1 promoter hypermethylation and were considered “screen-negative”. The remaining MMR-deficient cases (n = 16) were considered “screen-positive” and were referred for genetic counselling and testing. Germline PVs were identified in 12/16 (75%). One additional PV was identified in a screen-negative individual who was independently referred to the Genetics service. This corresponds to an overall LS frequency of 5.0% among unselected women with EC, and 6.4% among women diagnosed under age 70 years. Our algorithm detected MMR gene pathogenic variants in 4.6% and 6.2% of unselected individuals and individuals under age 70 years, respectively. Four germline PVs (30.8%) were identified in individuals who did not meet any traditional LS screening criteria. Conclusions: Universal IHC screening for women with EC is an effective and feasible method of identifying individuals with LS in a Canadian context.

## 1. Background

Endometrial cancer (EC) represents the most common gynecological malignancy diagnosed in Canadian women with a lifetime prevalence of approximately 2–3% [1]. It has been estimated that between 2% and 6% of ECs are due to Lynch syndrome (LS); a dominantly inherited cancer predisposition syndrome associated with an increased lifetime risk of developing a wide range of malignancies including colorectal, endometrial, gastric, ovarian, pancreatic, upper urinary tract, and brain cancers [2,3,4,5,6]. Colorectal cancer (CRC) and EC represent the two most common tumour types seen in individuals with LS with lifetime risks estimated to be in the range of 10–47% and 27–71%, respectively [7,8,9,10,11,12,13,14].

LS is caused by germline PVs in any one of five genes: *MLH1*, *MSH2*, *MSH6*, *PMS2*, and *EPCAM* [15]. These genes each have an effect on the DNA mismatch repair (MMR) pathway which identifies and corrects errors in the DNA sequence generated during DNA replication [15]. In this pathway, the MSH2 and MSH6 proteins function together as a heterodimer, as do the MLH1 and PMS2 proteins. Heterozygous germline PVs resulting in deficient production of the MLH1, MSH2, MSH6, and/or PMS2 proteins leads to LS^15^ as well as truncating terminal deletions of the *EPCAM* gene, which cause inactivation of the *MSH2* gene by hypermethylation of its promoter region [16].

## 2. Identifying Individuals with Lynch Syndrome: Old and New Methods

Several strategies for identifying individuals with LS have been proposed and tend to include a combination of clinical/family history-based criteria (Amsterdam I/II criteria [17], Revised Bethesda guidelines [18]), tumour tissue testing (IHC, microsatellite instability), algorithm-based prediction models (PREMM_5_, MMRpro), or universal germline genetic testing. Each of these methods has its own benefits and drawbacks: Screening by clinical/family history criteria or algorithm-based prediction models is inexpensive but relies heavily on the availability of accurate information necessary to assess risk. Possible barriers to obtaining such information may include a lack of clinic time to collect information, unavailability of clinical documentation, poor patient knowledge of family medical history, small family size, and lower-penetrance genetic variants [19,20,21,22]. This can lead to a significant number of patients with LS remaining undetected in the absence of a strong clinical suspicion. In contrast, screening strategies that utilize universal tumour testing either by IHC and/or microsatellite instability (MSI) have been shown to have improved sensitivity for identifying individuals with LS [19,20,21,22]. Although universal screening is more resource-dependent than clinical/family history-based approaches, additional clinical information gained from tumour testing such as the presence of MSI or specific absent protein expression can influence treatment and/or surgical decision-making and improve access to germline genetic testing for at-risk individuals [19,20,21,22].

Aberrant MMR protein expression is the hallmark of LS, making IHC screening an ideal strategy for assessing risk for LS via the expression of functional protein products of *MLH1*, *MSH2*, *MSH6*, and *PMS2* in tumour tissue [23]. Absent expression of one or more MMR proteins in tumour tissue can identify individuals at higher risk to have a germline PV. Improving the timely identification of individuals with LS is important for medical management as it allows for appropriate genetic counselling regarding additional cancer risks, surveillance recommendations, as well as risk-reducing options. Furthermore, once a pathogenic LS variant is identified in an affected individual, cascade testing of at-risk family members can be offered to identify which individuals would benefit from increased surveillance for LS-associated cancers, and in whom increased screening is unnecessary [4].

Increasingly, a consensus across several studies has indicated a gradual shift towards universal screening approaches for identifying individuals with LS [15]. To date, several American publications have concluded that universal screening of newly diagnosed CRCs by IHC is cost-effective from a US healthcare perspective [24,25]. Additionally, in their 1998 study, Vasen et al. found that using MMR germline status to determine screening strategies for CRC was cost-effective in a universal healthcare system [26]. A few studies have also suggested that universal IHC and/or MSI screening in newly diagnosed EC has higher sensitivity than both the revised Bethesda and Amsterdam II criteria [4,27].

To date, few published guidelines exist in Canada recommending routine screening of EC via universal tissue-based approaches despite the high risk of EC in LS patients and the similar proportions of EC and CRCs that are attributed to LS. In their 2018 practice guidelines for endometrial neoplasms, the National Comprehensive Cancer Network (NCCN) recommend universal screening of endometrial tumours for loss of MMR protein expression [28]. Recently, Lee et al. evaluated the efficiency of IHC screening for LS in a Vancouver population, and found that the implementation of universal screening increased efficiency in detecting LS. They suggested that secondary screening, including BRAF and MLH1 promoter hypermethylation testing, could further increase cost-effectiveness in a universal healthcare system [29]. Our goal was to therefore contribute to the existing literature regarding the feasibility and performance of a universal IHC screening program for all newly diagnosed ECs within a single Canadian university hospital setting.

## 3. Methods

In October 2015, a universal IHC screening program to identify MMR-deficient ECs was implemented by Gynecologic Pathology at the McGill University Health Centre (MUHC) as part of routine clinical care. The aim of this screening program was to improve ascertainment of women with LS presenting with EC and to assess the feasibility of a long-term IHC screening program. This retrospective health records research study comprised an analysis of all newly diagnosed ECs screened from 1 October 2015 to 31 December 2017. Following MUHC institutional review board approval, data were collected on the 261 ECs screened for MMR deficiency during this period. All consultation cases received from outside institutions where the patient was ultimately treated outside of the MUHC were excluded from analysis. Patients diagnosed with recurrent EC during the study period whose initial diagnosis was prior to the study start date were excluded, as well as patients with previous IHC testing or a known diagnosis of LS.

IHC analysis was performed by Gynecologic Pathology at the MUHC either at the time of endometrial biopsy or at the time of surgical resection. One representative section was selected for each patient. Antibodies used were for MLH1 (1:30, clone G168-15, mouse monoclonal: BioCare Medical, Pacheco, CA, USA), MSH2 (predilute, clone G219-1129, mouse monoclonal: CellMarque, Rocklin, CA, USA), MSH6 (1:100, clone EPR3945, rabbit monoclonal: ABCAM, Toronto, ON, Canada) and PMS2 (predilute, clone EPR3947, rabbit monoclonal: CellMarque, Rocklin, CA, USA) using the Benchmark Ultra platform (Roche, Indianapolis, IN, USA) Staff pathologists at the MUHC evaluated staining in the tumor cells using endometrial stroma, non-neoplastic endomyometrium, and lymphocytes as positive internal controls, together with on-slide positive and negative external controls.

Sectioned endometrial tumours with deficient MLH1/PMS2 protein expression were subsequently sent to the molecular pathology laboratory for MLH1 promoter methylation analysis. MLH1 promoter methylation was determined using the protocol described by Weisenberger DJ et al. [30], after complete bisulfite conversion of GC-rich DNA using the EZ DNA Methylation-GoldTM Kit (Zymo Research, Irvine, CA, USA). All patients with aberrant MMR protein expression (excluding cases with MLH1 promoter hypermethylation) were then referred by the treating Gynecologist Oncologist to the Medical Genetics service and offered genetic counselling and germline genetic testing. Germline testing was performed using a hybrid capture-based protocol and sequenced on an Illumina MiSeq (Illumina, San Diego, CA, USA) The following transcripts were used in the analysis: MLH1 (NM_000249.3), MSH2 (NM_000251.2), MSH6 (NM_000179.2), PMS2(NM_000535.5) (NR_003085.2), EPCAM (NM_002354.2). Nucleotide numbering was based on Gen Bank accession numbering of these genes. Additional genetic testing may have been ordered for a subset of patients depending on their personal and family histories. Any genetic testing results outside of germline LS PVs are not reported here.

### 3.1. Data Collection

A query of the MUHC Department of Pathology’s laboratory information system was conducted by searching for all laboratory accession numbers of specimens with endometrial carcinoma, diagnosed from 01 October 2015 to 31 December 2017. These accession numbers were then manually searched to obtain the associated medical record number (MRN). Samples with no associated MRN were excluded (i.e., patients not treated at the MUHC). Samples that were classified as endometrial intraepithelial neoplasia (EIN) were excluded, but any cases of EIN that were described as “borderline carcinoma” or for which the pathologist was unable to rule out an early carcinoma were included. The resulting list of MRNs was cross-checked with a local database kept within the MUHC Division of Medical Genetics to ensure a complete dataset. The MRNs were then used to access each patient’s electronic health record (EHR).

**Figure 1:**
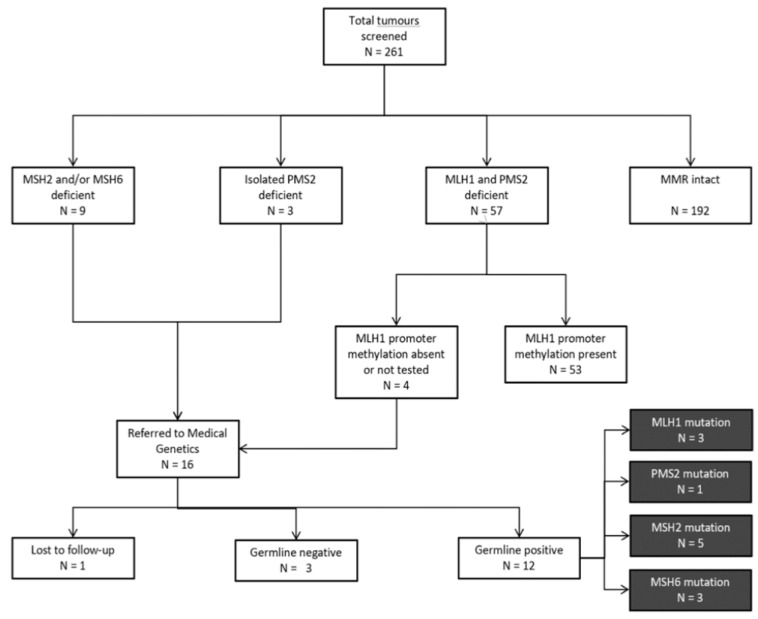
Flow diagram of the analytical strategy and main results of this study. Note: one additional *PMS2* germline mutation was identified in a patient that was screen-negative. This patient was not included in calculations of screen efficacy but was included in calculations of estimated germline mutation rate among unselected endometrial cancer patients.

The following information was extracted from the EHR for all patients: age at primary diagnosis; date of primary diagnosis, biopsy, and surgery; histological classification and tumour grade; histology comments; and results of IHC staining for MMR proteins. For patients that had absent MLH1 expression, MLH1 promoter methylation results were accessed. All patients with intact IHC staining, or absent MLH1 and PMS2 expression with somatic MLH1 promoter methylation were considered “screen-negative” (Figure 1). For “screen-positive” patients referred to Medical Genetics due to (i) absent MLH1/PMS2 expression with absent promoter methylation; (ii) absent MSH2 and/or MSH6 expression; or (iii) isolated absent PMS2 expression, information regarding ethnicity, family history of LS-associated cancers, genetic testing ordered, and outcome of genetic testing was extracted from genetics consult notes and pedigrees via the EHR.

### 3.2. Data Analysis

The performance of our screening algorithm was compared to traditional history-based screening criteria (Amsterdam II, Revised Bethesda guidelines) and to PREMM_5_, a statistical risk assessment model used to identify individuals at increased risk of LS [31]. In the PREMM_5_ model, a risk score is generated based on an individual’s personal and family history of LS-associated cancers, and a risk cut-off of 2.5% or greater is used to identify those patients for whom an evaluation for LS is recommended [31]. For each model, LS-associated cancers were categorized according to the screening criteria being used: for Amsterdam II criteria, these cancers are listed as CRC, cancer of the endometrium, small bowel, ureter, or renal pelvis; for revised Bethesda criteria these cancers are listed as colorectal, endometrial, gastric, small bowel, ovarian, pancreatic, ureter, renal pelvis, biliary tract, and brain (glioblastoma) tumors, sebaceous gland adenomas and keratocanthomas [17,18]. Additionally, descriptive and quantitative summary statistics were gathered to compare individuals with germline PVs to the “likely negative” cohort. The latter group of patients included all individuals who were either “screen-negative” by our protocol or “screen-positive” individuals who had negative germline MMR gene testing. These groups were compared using Fishers exact test and the Chi-square test using SPSS (IBM, Armonk, NY, USA).

The number of LS cases detected per tumour screened at our institution was calculated. Our findings were compared with published data to determine whether universal screening at the MUHC had similar results to previously published literature that recommends universal screening (IHC ± MSI) for CRC and/or EC. Results are likely low-biased due to the assumption that all individuals who meet traditional screening criteria would be referred to Medical Genetics by their treating physician, though the accuracy of this assumption cannot be assessed in this study.

## 4. Results

**Figure 2:**
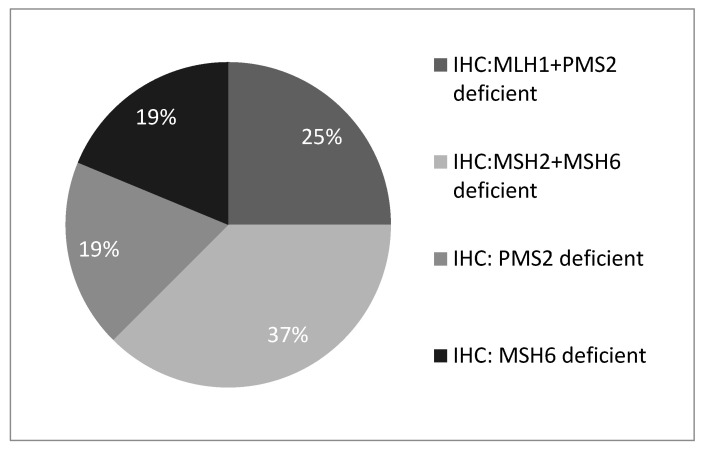
Breakdown of all screen-positive individuals by MMR protein deficiency. Note: MLH1 + PMS2 categories refer to patients with absent MLH1 + PMS2 expression on IHC and absent MLH1 promoter methylation.

A total of 261 tumour samples were screened for MMR deficiency from 261 unselected women with newly diagnosed EC aged 30–91 years at the time of diagnosis. Amongst all tumours tested, 192 (74%) were classified as “screen-negative” at the first step of the protocol due to intact MMR protein expression (Figure 1). Of the remaining 69 tumours with deficient MMR protein expression, 57 (83%) were further classified as “screen-negative” due to the presence of somatic MLH1 promoter methylation. A total of 16 “screen-positive” tumours were identified in 16 women aged 36–70 years at diagnosis (Figure 1). Of these, 4 (25%) showed MLH1 and PMS2 protein deficiency, 6 (37%) showed MSH2 and MSH6 deficiency, 3 (19%) showed isolated PMS2 deficiency, and 3 (19%) showed isolated MSH6 deficiency (Figure 2).

**Figure 3:**
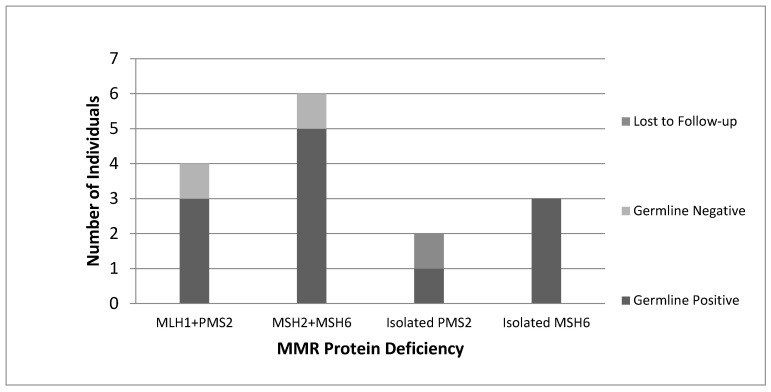
(Right): Results of genetic testing for all screen-positive individuals. Note: MLH1 + PMS2 categories refer to patients with absent MLH1 + PMS2 expression on IHC and absent MLH1 promoter methylation.

All women with “screen-positive” tumours identified by our protocol were subsequently referred by their Gynecologist Oncologist to the Medical Genetics service and offered germline genetic testing. Of the 16 women referred for genetic counselling, 15 consented to genetic testing, with 1 patient lost to follow-up. A total of 12 germline MMR PVs were identified (Figure 3). Overall, 1 germline PV was detected per 22 tumours screened (4.6%).

### 4.1. Performance of Universal Screening vs. Alternative Screening Methods

A total of 12 germline PVs were identified by our universal screening program; 3 in *MLH1*, 1 in *PMS2*, 5 in *MSH2,* and 3 in *MSH6*. One additional germline *PMS2* PV was identified in a screen-negative patient (MLH1/PMS2 deficient, MLH1 methylation present) who was referred to genetics outside of this protocol, based on family history. This corresponds to an overall LS prevalence of 5% in an unselected series of women with EC treated at a single hospital centre, and a screen detection rate of 4.6%. Twelve of the 13 women (92.3%) with MMR germline PVs met the 2.5% assessed risk cut-off given by the PREMM_5_ model, 9 (69%) met the revised Bethesda guidelines, and only 2 (15%) women met the Amsterdam II criteria (Table 1). Women seen for genetic counselling were of mixed ancestry including French Canadian, South American, South Asian, Northeast European, Middle Eastern, and Southwestern European descent. Detailed family history information for patients evaluated in Medical Genetics is available in the supplemental materials (Table S1).

### 4.2. Characteristics of Germline-Positive vs. Likely Negative Cohorts

**Figure 4:**
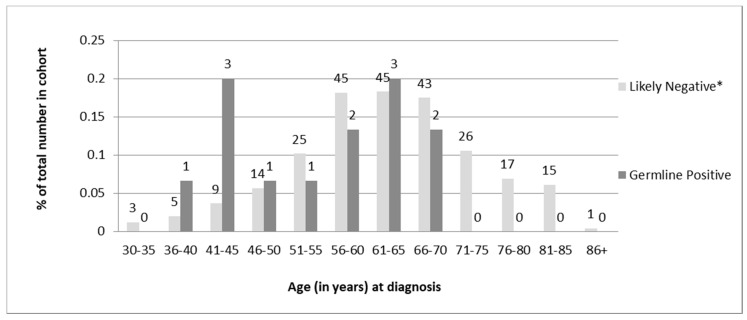
Comparison of the distribution of age at diagnosis of EC between likely negative and germline-positive cohorts. Actual N for each group is shown above each column. Comparison between these two groups using Fisher’s exact test revealed a statistically significant difference (*p* = 0.02). Note: Patients who were screen-negative based on the protocol and patients whose germline testing results were negative were considered likely negative for these analyses.

Of the 13 individuals found to carry germline PVs, 6 (46%) were diagnosed under age 50, and all were under 70 years of age (Table 1). Chi-square analysis of the distribution of age at diagnosis revealed a statistically significant difference (*p* = 0.02) in the age of diagnosis for germline-positive vs -negative women. However, there was a sizeable overlap in the ages of diagnosis between the two groups (Figure 4).

**Figure 5:**
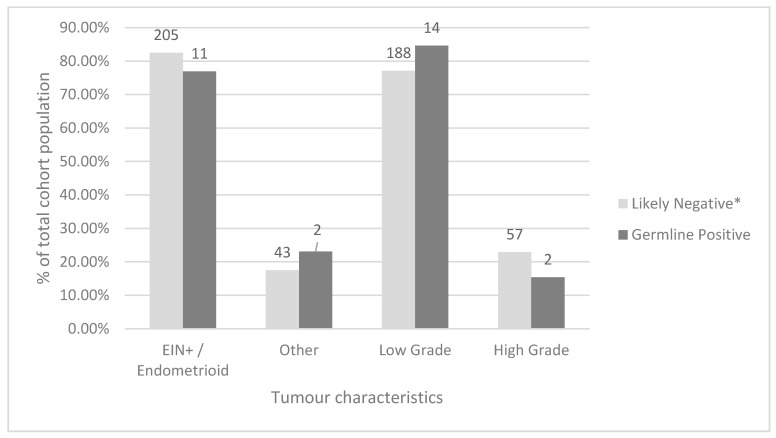
Comparison of tumor pathology and grade at time of diagnosis between screen-negative and germline-positive cohorts. EIN+ = EIN with pathological features bordering on early carcinoma. Actual N for each group is shown above each column. * Patients who were screen-negative based on the protocol and patients whose germline testing results were negative were considered likely negative for these analyses.

Amongst all screened tumours, the most common histological type was endometrioid (206, 78.9%), followed by serous (25, 9.6%). Low grade (classified as FIGO grade 1 or 2) carcinomas were more common than high grade (classified as higher than FIGO grade 2) carcinomas (203 or 77.8% and 58 or 22.2%, respectively). When comparing women who were screen-negative by our protocol to women with germline MMR PVs using Fisher’s exact test, there was no statistically significant difference between tumour histology or FIGO grade between these groups (*p* = 0.59 and 0.42 respectively) (Figure 5). Additional data comparing the total study, screen-negative, screen-positive, germline-positive, Bethesda-positive, and Amsterdam-positive cohorts are available in Supplemental Table S2.

## 5. Discussion

The results of this single-centre Canadian study demonstrate that within a multidisciplinary model of care that includes Pathology, Gynecologic Oncology, and Medical Genetics, universal IHC screening of newly diagnosed ECs is a highly feasible and effective strategy for identifying women with LS, with very few individuals lost to follow-up.

### 5.1. Comparison of LS Screening Protocols

None of the screening models, including our protocol, was able to identify all 13 of the individuals found to have germline MMR PVs. Notably, the 12 women identified by the PREMM_5_ model were not the same 12 that were identified by our universal screening algorithm. The one individual missed by our model was found to carry a germline *PMS2* PV outside of the study protocol. She had been categorized as “screen-negative” by our model based on MLH1/PMS2 protein deficiency in her tumour and presence of MLH1 promoter methylation. This individual was referred for genetic counselling outside of the study protocol, based on a family history of renal cancer. Interestingly, the one individual missed by the PREMM_5_ model was found to have a germline *MLH1* PV by our protocol; a gene associated with comparatively higher overall penetrance than *PMS2*. This is in keeping with published literature that suggests that individuals with MSH6 and PMS2 PVs are more likely to be missed by an IHC screening protocol [32,33]. This underscores the importance of using IHC as a screening test. While our protocol did not include universal germline testing of the “screen-negative” group, a study by Ferguson et al., which compared the performance of universal germline testing in a cohort of EC patients to that of tumour-based testing approaches (MSI and IHC), did not identify any additional germline PVs amongst EC patients with intact IHC and MSS tumours [6]. Interestingly, 25% of women enrolled in the study, the majority of whom had intact IHC, declined germline genetic testing [6], suggesting that perhaps a subset of women felt sufficiently reassured by the normal results of tumour-based testing. Ultimately, as seen in our cohort, a negative IHC screen should not eliminate LS from consideration, particularly in individuals with clinical suspicion of LS based on family history. The strength of a universal screening approach is, however, in improving identification of individuals with LS who may not otherwise meet traditional clinic-based criteria.

Although discordance between IHC staining results and MMR germline status has been described in the literature [32], we found that amongst the “screen-positive” individuals who were offered germline testing, genetic test results were concordant with IHC results for 92% (12/13) of germline-positive individuals. This concordance underscores the additional clinical utility of universal IHC screening as the only LS screen that can be used to guide genetic testing and in some cases inform chemotherapy treatment [34,35]. Universal IHC screening for LS can also assist with genetic assessment and testing of both MMR-screen-positive and screen-negative individuals with a high suspicion of an inherited cancer syndrome by helping to direct whether testing should be restricted to MMR genes versus a larger panel encompassing other cancer predisposition genes. This is particularly useful in a Canadian healthcare setting where core hereditary cancer gene panels can be more readily developed by in-house molecular laboratories, thereby reducing the need for out-of-country send-outs for commercial third-party testing.

### 5.2. Implications of Tumour Grade, Histology, and Age at Diagnosis on LS Risk

Overall, low-grade tumour histology was observed in more than three-quarters of tumours screened in our study which is consistent with previously published literature on a similar Canadian cohort [6]. When comparing the germline-positive and likely negative groups in our study population, no statistical difference was observed for tumour histology or tumour grade (*p* = 0.59 and *p* = 0.42, respectively) suggesting that tumour morphology is not a reliable clinical indicator of a patient’s likelihood to have a germline MMR PV. This is in keeping with other data published in a Canadian cohort [6].

In terms of the overall distributions of ages at diagnosis; some differences were observed between the two groups. None of the 13 individuals with germline MMR PVs were over the age of 67 at diagnosis though less than half (46%) were diagnosed prior to age 50. Although the data are skewed towards younger age of diagnosis for germline-positive women compared to screen-negative women, there is a significant overlap in the ages of diagnosis between the two groups. This provides evidence for recommending a screening cut-off for patients older than 70 years at diagnosis rather than the traditionally used age cut-off of 50 years proposed by the revised Bethesda guidelines [18].

## 6. Discussion of Limitations

Limitations of this study include the overall small sample size, limited to a single university hospital centre, as well as the exclusion of endometrial intraepithelial neoplasia (EIN) cases from the screening protocol. Additionally, given that screen-negative patients were not referred to the Medical Genetics service under our protocol, data on the family histories, ethnicities, and germline MMR status of the “screen-negative” cohort could not be assessed. In the absence of complete clinical and family history information and germline MMR test results for the entire cohort, positive predictive value, negative predictive value, and screen sensitivity cannot be accurately assessed for any of the screening modalities compared in this study, nor can an overall cost–benefit ratio of universal IHC. The exclusion of EIN from screening protocols is common, but data have recently emerged showing the presence of MMR PVs in a small subset of individuals with EIN [36]. As such, this population could be considered for universal IHC screening programs in the future, though inclusion of all EIN in this study would have likely decreased the efficiency of the screen, requiring more tumours to be screened for every germline PV identified. Of note, we did identify one *MSH6* germline PV amongst our EIN tumours with “borderline carcinoma” (n = 9), which could add evidence in support of inclusion of EIN in universal screening protocols in the future.

The overall prevalence of LS in our cohort was 5.0%, but this figure is likely low-biased due to the possibility for germline PVs to be missed by IHC, particularly in *MSH6* or *PMS2* [32,33]. This was illustrated by the additional germline *PMS2* PV identified in a “screen-negative” individual with MLH1/PMS2 protein deficiency and positive MLH1 promoter methylation in her tumour. Our observed MMR mutation prevalence falls within the published range of 2–6% amongst unselected ECs [2,3,4,5,6] but is nonetheless at the higher end of the range compared to most universal screening studies employing either MSI and/or IHC [4,27,37,38]. This could be due to differences in the types of PVs identified by MSI vs. IHC, a higher-than-average rate of LS among our study cohort (the MUHC is a university teaching hospital, perhaps serving a higher-risk population), an increase in the number of individuals missed by other two-step screening protocols (MSI then IHC, or IHC then MSI), differences in IHC efficacy between centers, or the fact that 16 out of 17 patients referred for genetic counselling in our study ultimately consented to genetic testing, as compared to higher rates of drop-out/decline of testing in other published studies [29,37,38].

## 7. LS Screening in a Canadian Context

We propose that in a single-payer healthcare system, using IHC to screen all newly diagnosed ECs in individuals under the age of 70 years could provide a higher cost–benefit ratio than universal screening with no upper age limit. In this study, restricting our protocol to individuals under the age of 70 years would decrease both the number of tumours screened and the number of tumours requiring MLH1 promoter methylation analysis by 26% and 28%, respectively, while still identifying all individuals found to have a germline PV. Using a screening cut-off age of 69 years at diagnosis increases our screening algorithm’s detection rate to 6.2% and the overall MMR germline-positive rate in this cohort to 6.4%. This LS prevalence is higher than that cited by published studies which recommend universal IHC screening of CRCs [25,39,40,41,42,43,44,45], and similar to the LS prevalence among women with EC observed in an Ontario population [6]. It therefore follows that universal IHC screening of newly diagnosed ECs should be implemented in most Canadian hospital centres as part of routine clinical care, especially in women diagnosed under the age of 70 years.

Universal IHC screening for newly diagnosed CRCs under the age of 70 years has been reported in the literature as one of the most cost-effective screening methods for LS [25], and so it follows with the similar incidence of LS among ECs that this strategy could also be highly cost-effective in an EC cohort. It is important to note, however, that other studies have reported MMR germline PVs in women diagnosed as late as in their 70s and 80s [37,38,45], and so using an age cut-off of 70 years could fail to identify a small proportion of older women with LS. We therefore recommend that gynecologic oncologists ask about LS-related cancers in a patient’s family history and refer to Genetics in the event of a positive family history, irrespective of a patient’s age.

Universal IHC screening in newly diagnosed ECs stands to address issues related to LS ascertainment in the Canadian healthcare system. It has been suggested that IHC screening, particularly for ECs, is either unavailable or under-accessed by physicians in many Canadian healthcare settings [19]. A survey of Canadian pathologists and genetic counsellors cited lack of an interdisciplinary approach, lack of funding, and a lack of genetic counsellors as key barriers preventing the establishment of universal screening programs in hospital centres across Canada [46]. Additionally, with regards to algorithm-based screening such as PREMM_5_, family history information may be difficult to obtain and analyse in busy clinical practice both due to physician time restraints and limited patient knowledge [47]. Universal IHC screening for ECs diagnosed under the age of 70 years eliminates the need to record and analyse family history information at the time of diagnosis and creates a standardized procedure for physicians to follow. The removal of barriers imposed by clinical and family history-based ascertainment has the potential to minimize the number of individuals and families with LS who would otherwise be missed. This improved ascertainment would then in turn both improve survival in newly ascertained individuals with LS and decrease healthcare costs by diagnosing cancers earlier or preventing them altogether.

## 8. Conclusions

We conclude that universal IHC screening among women with EC is an effective and efficient method to improve ascertainment of Canadian women and their families with LS. Our screening protocol performed as well as the PREMM_5_ model and better than both the Revised Bethesda Guidelines and Amsterdam II criteria. Advantages of IHC screening over these other approaches include the additional clinical information provided by IHC and the removal of barriers implicit in family history-based testing.

The germline MMR prevalence rate among unselected women with EC in our center was 5.0%, and 6.4% among women diagnosed prior to age 70 years. These rates are higher than the LS prevalence rates cited by published studies which recommend universal IHC screening in CRC. It therefore follows that universal IHC screening should be incorporated into the routine clinical care of Canadian women diagnosed with EC. In a single-payer healthcare system, IHC screening of all endometrial tumours diagnosed under the age of 70 years could be a cost-effective alternative to universal IHC screening, but further research, perhaps in a multi-centre Canadian cohort, is needed to assess this assumption.

## Figures and Tables

**Table 1:** Clinical information for germline-positive individuals.

ID#	Mutation	Age	Ethnicity	AII Met?	RB Met?	PREMM_5_ Prediction
155	MSH6 c.1139_1143delATGAG	42	Peruvian	N	Y	10.50%
80	MSH6 c.4001G>A	55	Spanish	N	N	5%
110	MSH6 c.2300C>T	58	Polish, German, and English	N	N	3.20%
256	PMS2 exon 6-8 deletion	62	French Canadian and Anglo-Saxon	N	Y	3%
239	MSH2 exon 7 deletion	48	Syrian	Y	Y	>50%
158	MSH2 c.882dupT	36	Turkish	N	Y	3.70%
55	MSH2 c.1786_1788delAAT	61	French Canadian and Irish	N	Y	4.10%
223	MSH2 c.998G>A	41	Filipina	N	Y	3.30%
29	MSH2 c.1277-11C>G ^a^	58	Portuguese	N	N	2.50%
191	PMS2c.137G>T ^b^	66	French Canadian and Irish	N	Y	2.80%
162 ^c^	MLH1 c.2195_2198dupAACA	47	French Canadian	Y	Y	49.40%
99	MLH1 c.454-13A>G	44	Portuguese	N	Y	5.30%
254	MLH1 del exon 6	65	Sri Lankan	N	N	2.30%

^a^ This variant was classified as a variant of uncertain significance (VUS). It is included in this table because this patient’s MSH2 expression was absent on IHC; ^b^ This individual was screen-negative by our protocol (promoter hypermethylation). She was independently referred to genetics based on family history; ^c^ Patient 162 was aware of a diagnosis of LS in her father prior to meeting genetics but was ascertained by this screening program and is therefore included. Legend: Age—years old at diagnosis; AII—Amsterdam II criteria; RB—Revised Bethesda criteria; PREMM—risk to carry germline MMR mutation calculated by PREMM_5_ algorithm.

## Data Availability

Data available on request due to restrictions. The data presented in this study are available on request from the corresponding author. The data are not publicly available due to privacy concerns.

## Supplementary Materials

The following are available online at https://www.mdpi.com/1718-7729/28/1/52/s1, Table S1: Medical and family history information and mutations identified in germline-positive individuals; Table S2: Comparison of age at diagnosis, tumour histology, and tumour grade across subgroups.
