# Supplementary material for: Mismatch Repair Universal Screening of Endometrial Cancers (MUSE) in a Canadian Cohort
Source: Curr Oncol. 2021 Jan 15;28(1):509–22. doi: 10.3390/curroncol28010052 (PMC7903281; doi:10.3390/curroncol28010052)
Supplement: Supplementary file 1 [file curroncol-28-00052-s001.pdf]

Current Oncology

MDPI

Supplementary Materials

Mismatch Repair Universal Screening of Endometrial Cancers (MUSE) in a Canadian Cohort

Jessica Lawrence [1,*], Lara Richer [2], Jocelyne Arseneau [2], Xing Zeng [3], George Chong [4], Evan Weber [5],William Foulkes [1,5,6], Laura Palma [1,5]

Table S1. Medical and family history information and mutations identified in germline positive individuals.

ID#	Ethnicity	Age at Diagnosis	Mutation	Histology	Tumour grade	Family history of cancer
155	Peruvian	42	MSH6 c.1139_1143delATGAG	EIN+	1	Mother: osteosarcoma age 48 years.<br>Two sisters: endometrial cancer ages 44 and 49 years.<br>Maternal uncle: lung cancer age 60 years.<br>Maternal aunt: thyroid cancer in her 60s.
80	Spanish	55	MSH6 c.4001G>A	M	2 to 3	Sister: endometrial cancer age 52 years.
110	Polish, German, and English	58	MSH6 c.2300C>T	E	1	Father: kidney cancer age 64 years, prostate cancer age 70 years.<br>Paternal grandmother: breast cancer, unknown age.<br>Paternal first cousin once-removed: endometrial cancer age 50 years.<br>Maternal uncle: leukemia age 60 years.<br>Maternal first cousin (male): thyroid cancer in his 50s.
256	French Canadian and Anglo-saxon	62	PMS2 exon 6-8 deletion	E	2	Mother bile duct cancer age 73 years. Also has NF1.<br>Sister: ovarian cancer age 48 years. Also has NF1.<br>Sister: breast and bone cancer age 60 years. Also has NF1.<br>Sister: colorectal polyps age 59 years.<br>Father: lung cancer age 69 years.<br>Paternal aunt: breast cancer in her 60s.
239	Syrian	48	MSH2 exon 7 deletion	E	2	Mother: colorectal cancer in her 50s.<br>Sister: colorectal cancer age 36 years.
158	Turkish	36	MSH2 c.882dupT	E	1	Father: prostate cancer in his 60s.
55	French Canadian and Irish	61	MSH2 c.1786_1788delAAT	E	2	Father: colorectal cancer age 50 years.<br>Brother: Hodgkin's lymphoma age 27 years.
223	Filipina	41	MSH2 c.998G>A	E	2	Mother: possible endometrial cancer in her 50s.<br>Maternal first cousin: either uterine fibroids or cancer at unknown age.<br>Maternal second cousin (female) with breast cancer.
29	Portuguese	58	MSH2 c.1277-11C>G *	M	2	Two sisters: breast cancer ages 66 and 70 years.<br>Maternal first cousin: stomach cancer in her 60s.
191	French Canadian and Irish	66	PMS2c.137G>T **	E	3	Father: kidney cancer 68 years<br>Mother: ureter cancer 90 years.<br>Brother: lung and throat cancer age 60 years.
162	French Canadian	47	MLH1 c.2195_2198dupAACA	E	1	Father: colorectal cancer age 32 years.<br>Brother: colorectal cancer in his 50s.<br>Sister: colorectal cancer age 40 years and endometrial cancer age 50 years.<br>Paternal uncle: stomach cancer in his 50s.

99	Portugues e	44	MLH1 c.454-13A>G	E	2	Paternal aunt: lung cancer age 67 years. Paternal aunt ("A"): breast cancer in her 40s "A"'s son: colorectal cancer age 45 years. Father: colorectal cancer age 54 years, kidney cancer age 61 years, and prostate cancer age 63 years. Paternal grandfather: carcinoid appendix age 58.
254	Sri Lankan	65	MLH1 del exon 6	E	1	None

EIN + = EIN with pathological features bordering on early carcinoma. E = Endometrioid adenocarcinoma. M = Mixed Histology. S = Serous carcinoma. NF1 = Neurofibromatosis type 1. * This variant was classified as a variant of uncertain significance. It was counted as pathogenic for the purposes of this study because MSH2 expression was also absent on IHC. ** This individual was "screen negative" by our protocol due to the presence of promoter hypermethylation. He was referred to genetics outside of this study's protocol, and a PMS2 germline mutation was identified. Note: Patient 162 was aware of a diagnosis of Lynch syndrome in her father prior to meeting genetics but was ascertained by this screening program and is therefore included.

Table S2. Comparison of age at diagnosis, tumour histology, and tumour grade across subgroups.

	**Total Cohort**	**Screen Negative Cohort**	**Screen Positive Cohort**	**Germline Positive Cohort***	**Patients who meet revised Bethesda Guidelines****	**Patients who meet Amsterdam II Criteria****
N	261	245	16	15	9	2
Median age at diagnosis in years	63	63	56	56.5	47	47.5
Mean age at diagnosis in years	62.5	63.0	53.9	53.6	49.7	47.5
Age Range (years)	30-91	30-91	36-70	36-66	36-66	47-48
Histology: EIN+	9 (3.4%)	8 (3.3%)	1 (6.3%)	1 (6.7%)	1 (11.1%)	0
Histology: Endometrioid	206 (78.9%)	194 (79.2%)	12 (75.0%)	11 (73.3%)	8 (88.9%)	2 (100%)
Histology: Serous	25 (9.6%)	24 (9.8%)	1 (6.3%)	1 (6.7%)	0	0
Histology: Other	8 (3.1%)	8 (3.3%)	0	0	0	0
Histology: Mixed	13 (5.0%)	11 (4.5%)	2 (12.5%)	2 (13.3%)	0	0
Grade: Low (grade 1)	152 (58.2%)	145 (59.2%)	7 (43.8%)	6 (40.0%)	3 (33.3%)	1 (50%)
Grade: High (above grade 1)	109 (41.8%)	100 (40.8%)	9 (56.3%)	9 (60.0%)	6 (66.7%)	1 (50%)

*Patients 150 and 198 were lost to follow-up and were considered germline positive for this table. These patients were considered positive due to the high correlation between deficient PMS2 staining on IHC and germline PMS2 mutations. **Total number of patients assessed by Bethesda and Amsterdam criteria was 13.
